# Sensitivity of International Classification of Diseases codes for hyponatremia among commercially insured outpatients in the United States

**DOI:** 10.1186/1471-2369-9-5

**Published:** 2008-06-18

**Authors:** Alisa M Shea, Lesley H Curtis, Lynda A Szczech, Kevin A Schulman

**Affiliations:** 1 Center for Clinical and Genetic Economics, Duke Clinical Research Institute, PO Box 17969, Durham, North Carolina, USA; 2 Duke Clinical Research Institute, Durham, North Carolina, USA; 3 Division of General Internal Medicine, Department of Medicine, Duke University School of Medicine, Durham, North Carolina, USA; 4 Division of Nephrology, Department of Medicine, Duke University School of Medicine, Durham, North Carolina, USA

## Abstract

**Background:**

Administrative claims are a rich source of information for epidemiological and health services research; however, the ability to accurately capture specific diseases or complications using claims data has been debated. In this study, the authors examined the validity of *International Classification of Diseases, Ninth Revision, Clinical Modification *(ICD-9-CM) diagnosis codes for the identification of hyponatremia in an outpatient managed care population.

**Methods:**

We analyzed outpatient laboratory and professional claims for patients aged 18 years and older in the National Managed Care Benchmark Database from Integrated Healthcare Information Services. We obtained all claims for outpatient serum sodium laboratory tests performed in 2004 and 2005, and all outpatient professional claims with a primary or secondary ICD-9-CM diagnosis code of hyponatremia (276.1).

**Results:**

A total of 40,668 outpatient serum sodium laboratory results were identified as hyponatremic (serum sodium < 136 mmol/L). The sensitivity of ICD-9-CM codes for hyponatremia in outpatient professional claims within 15 days before or after the laboratory date was 3.5%. Even for severe cases (serum sodium ≤ 125 mmol/L), sensitivity was < 30%. Specificity was > 99% for all cutoff points.

**Conclusion:**

ICD-9-CM codes in administrative data are insufficient to identify hyponatremia in an outpatient population.

## Background

Hyponatremia, defined as an abnormally low level of serum sodium, is the most frequently observed electrolyte disorder in the United States and is associated with significant morbidity and mortality in patients with heart failure [1,2], myocardial infarction [3,4], and liver cirrhosis [5,6], as well as in the hospitalized elderly population at large [7]. Among general acute care patients, the prevalence of hyponatremia is estimated to be approximately 1% [8,9]. However, much higher rates–ranging from 18% to 30%–have been observed among elderly nursing home residents [10] and in intensive care settings [11]. Little is known about the prevalence of hyponatremia in outpatient settings or in the general population.

Administrative claims data are a rich source of information for epidemiological and health services research. With increasing frequency, researchers are turning to administrative claims data to ascertain information about patient outcomes and hospital quality [12-19]. However, the ability to accurately capture specific diseases or complications using claims data has been a subject of considerable debate [20-24].

Most validation studies of diagnosis and procedure codes have relied on retrospective chart review as the source of comparative information. Using medical record review as the gold standard, Quan et al [25] found that the validity of *International Classification of Diseases, Ninth Revision, Clinical Modification *(ICD-9-CM) codes for invasive or major surgical procedures in inpatient discharge claims was high; however, codes for routine procedures were often inaccurate or incomplete. Geraci et al [26] used the same method to assess the validity of 30 ICD-9-CM codes for common in-hospital complications as observed in patient discharge records from nine hospitals. They found an overall sensitivity of 34% and a positive predictive value of 32%. A handful of studies have used laboratory information to validate administrative claims codes. Wei and Walsh [27] compared managed care claims data with laboratory results and found that less than 25% of female beneficiaries with a positive test for chlamydia were coded as such. In another study, researchers used clinical, radiological, and laboratory data to assess the validity of ICD-9 codes for the diagnosis of gout in an ambulatory managed care population and found a positive predictive value of 61% [28].

To our knowledge, there has been only one published study of the validity of ICD-9-CM codes for the diagnosis of hyponatremia. Movig et al [29] compared inpatient hospital discharge records with inpatient laboratory data and reported a sensitivity of 30% for even the strictest definition of hyponatremia (≤ 115 mmol/L). Positive predictive value for laboratory results showing serum sodium ≤ 135 mmol/L was 91.7%. The study did not address the validity of coding for hyponatremia outside the inpatient setting. Therefore, we sought to examine the validity of ICD-9-CM diagnosis codes for the identification of hyponatremia in an outpatient managed care population.

## Methods

### Data source

We used data from the National Managed Care Benchmark Database from Integrated Healthcare Information Services (IHCIS; Waltham, MA). The database includes complete medical and eligibility data from over 30 health plans covering more than 25 million lives in the United States. Outpatient laboratory data are available for approximately 10% of members, and outpatient pharmacy information is available for 90% of members. Laboratory tests performed during inpatient hospitalizations are not collected in the database. To protect member confidentiality, IHCIS removed all direct identifiers.

### Claim identification

We limited the analysis to claims filed in 2004 and 2005 for members aged 18 years and older. We obtained all claims for outpatient serum sodium laboratory tests performed between January 1, 2004, and December 31, 2005. Serum sodium values < 8 mmol/L were excluded (n = 1854) because these were considered data errors. All remaining values were ≥ 100 mmol/L. When multiple serum sodium tests were performed on the same day, we retained the highest value for the analysis. We also obtained all outpatient professional claims incurred in 2004 and 2005 with a primary or secondary ICD-9-CM diagnosis code of hyponatremia (276.1).

For each claim, members were required to have had continuous eligibility for at least 60 days before and 15 days after the claim date of service. Because this database represents a transient managed care population with a great deal of movement into and out of plans, we chose a 60-day period of observation prior to the serum sodium laboratory test so that we might still observe comorbid claims but not reduce the study population substantially by requiring a lengthy period of pretest eligibility. Multiple successive periods of eligibility, defined as an observed coverage end date followed immediately by a new coverage start date, were considered switches in insurance product and not a discontinuation of coverage; therefore, such changes were not considered an interruption in coverage but rather a single, continuous period of eligibility.

We excluded members with a professional claim for dialysis in 2004 or 2005. We also excluded members with serum/plasma triglycerides > 400 mg/dL as measured 15 days before or after the reference serum sodium date, due to the possibility that any observed changes in serum sodium were related to pseudohyponatremia. If blood glucose was > 300 mg/dL as measured 15 days before or after the reference serum sodium lab date, we adjusted the serum sodium laboratory result by a factor of 1.6 (adjusted value = original value + ([glucose – 100]/100) × 1.6) [30].

We reviewed inpatient, outpatient, and professional claims for evidence of underlying comorbid conditions within the 60-day period before through 15 days after the reference serum sodium date. Specifically, we searched for evidence of liver cirrhosis (ICD-9-CM code 572.4); congestive heart failure (428.0); nephritis, nephrotic syndrome, and nephrosis (580–589); and syndrome of inappropriate secretion of antidiuretic hormone (253.6). In addition, we identified comorbid conditions using the coding algorithms described by Birman-Deych et al [31] and Quan et al [32]. We searched all inpatient, outpatient, and professional claims for 60 days before through 15 days after the reference serum sodium date for evidence of cerebrovascular disease (362.34, 430.x-438.x), chronic obstructive pulmonary disease (416.8, 416.9, 490.x-505.x, 506.4, 508.1, 508.8), coronary heart disease (410.x-414.x, 429.2, V45.81), dementia (290.x, 294.1, 331.2), diabetes mellitus (250.x), hypertension (401.x-405.x, 437.2), kidney disease (403.01, 403.11, 403.91, 404.02, 404.036, 404.12, 404.13, 404.92, 404.93, 582.x, 583.0–583.7, 585.x, 586.x, 588.0, V42.0, V45.1, V56.x), metastatic carcinoma (196.x-199.x), peripheral vascular disease (093.0 437.3, 440.x, 441.x, 443.1–443.9, 47.1, 557.1, 557.9, V43.4), and rheumatic disease (446.5, 710.0–710.4, 714.0–714.2, 714.8, 725.x).

We also identified outpatient pharmacy claims for medications known to cause hyponatremia. We used National Drug Codes to identify claims for phenothiazines, selective serotonin reuptake inhibitors, thiazide diuretics, tricyclic antidepressants, prostaglandin synthesis inhibitors, desmopressin, oxytocin, opiate derivatives, chlorpropamide, clofibrate, carbamazepine, cyclophosphamide, or vincristine incurred 60 days before through 15 days after each outpatient serum sodium laboratory date [33].

Finally, for each member with an eligible outpatient serum sodium laboratory claim, we obtained all outpatient professional claims from 2004 or 2005 that did not include an ICD-9-CM diagnosis code for hyponatremia.

### Statistical analysis

We defined hyponatremia as serum sodium < 136 mmol/L [1,2,4,33-35]; however, we also performed validity analyses on three additional strata: serum sodium ≤ 133 mmol/L; ≤ 130 mmol/L; and ≤ 125 mmol/L. We used basic descriptive statistics to summarize demographic characteristics, comorbidities, and prescription drug claim information for members with serum sodium values indicating hyponatremia both with and without a corresponding outpatient professional claim for hyponatremia. We assessed differences between groups using χ^2 ^tests for categorical variables and Wilcoxon rank sum tests for continuous variables.

For all measurements of validity, we considered the laboratory result to be the gold standard of diagnosis and the outpatient professional claim to be the test. We defined sensitivity as the probability of a positive test–an outpatient professional claim with a primary or secondary ICD-9-CM diagnosis code of hyponatremia observed within 15 days before or after a serum sodium laboratory result indicating hyponatremia. We defined specificity as the probability of a negative test–an outpatient professional claim that did not include an ICD-9-CM code for hyponatremia or the absence of any outpatient professional claim within 15 days before or after a serum sodium laboratory result not indicating hyponatremia. Positive predictive value was the probability of a laboratory result indicating hyponatremia among positive outpatient professional claims; negative predictive value was the probability of a laboratory result not indicating hyponatremia among negative outpatient professional claims or in the absence of any outpatient claim.

Using all serum sodium laboratory claims indicating hyponatremia, we performed logistic regression analysis to explore the relationships between undocumented hyponatremia (serum sodium laboratory claim indicating hyponatremia, but no professional ICD-9-CM claim for hyponatremia) and members' demographic characteristics and comorbidities as observed within 60 days before through 15 days after the laboratory encounter.

We used SAS version 9.1.5 for all analyses (SAS Institute Inc, Cary, NC). The institutional review board of the Duke University Health System approved this study.

## Results

There were 1,901,254 eligible serum sodium laboratory claims in the study sample. Of these, 40,668 (2.1%) indicated hyponatremia (serum sodium < 136 mmol/L). Outpatient professional claims with an ICD-9-CM diagnosis code for hyponatremia were observed within 15 days before or after the hyponatremic serum sodium lab date for 1407 of these claims (3.5%) (Table 1).

**Table 1:** Sample characteristics*

Characteristic	Laboratory Claims Indicating Hyponatremia†			*p*||
		
	All Claims (n = 40,668)	Negative Test‡ (n = 39,261)	Positive Test§(n = 1407)	
Age, mean (SD), y	59 (15.7)	59 (15.7)	67 (12.3)	< 0.001
Female sex	24,694 (60.7)	23,769 (60.5)	925 (65.7)	< 0.001
Underlying conditions and comorbidities				
Cerebrovascular disease	1874 (4.6)	1721 (4.4)	153 (10.9)	< 0.001
Chronic obstructive pulmonary disease	4279 (10.5)	4020 (10.2)	259 (18.4)	< 0.001
Congestive heart failure	2742 (6.7)	2605 (6.6)	137 (9.7)	< 0.001
Coronary heart disease	5857 (14.4)	5560 (14.2)	297 (21.1)	< 0.001
Dementia	164 (4.0)	141 (3.5)	23 (1.6)	< 0.001
Diabetes mellitus	8342 (20.5)	8102 (20.6)	240 (17.1)	0.001
Hypertension	14,825 (36.5)	13,997 (35.7)	828 (58.8)	< 0.001
Kidney disease	1491 (3.7)	1405 (3.6)	86 (6.1)	< 0.001
Liver cirrhosis	26 (0.1)	24 (0.1)	2 (0.1)	0.24
Metastatic carcinoma	2325 (5.7)	2246 (5.7)	79 (5.6)	0.87
Nephritis, nephrotic syndrome, and nephrosis	1918 (4.7)	1800 (4.6)	118 (8.4)	< 0.001
Peripheral vascular disease	1613 (4.0)	1546 (3.9)	67 (4.8)	0.12
Rheumatic disease	1291 (3.2)	1254 (3.2)	37 (2.6)	0.24
Syndrome of inappropriate antidiuretic hormone	157 (3.9)	91 (2.3)	66 (4.7)	< 0.001
Medications known to cause hyponatremia	10,492 (25.8)	10,144 (25.8)	348 (24.7)	0.35

* Values are expressed as number (percentage) unless otherwise indicated.

† Hyponatremia was defined as serum sodium < 136 mmol/L.

‡ A negative test was defined as the presence of an outpatient professional claim with no ICD-9-CM code for hyponatremia or the absence of any outpatient professional claims within 15 days before or after the laboratory claim indicating hyponatremia.

§ A positive test was defined as the presence of an outpatient professional claim with a primary or secondary ICD-9-CM diagnosis code of hyponatremia observed within 15 days before or after the laboratory claim indicating hyponatremia.

|| *p*-Values for the comparison between the positive and negative test groups.

Mean age of members at the time of the laboratory claim indicating hyponatremia was 59 years, and over half of all claims were for women (61%). Hypertension was the most commonly identified comorbidity in this sample. Evidence of hypertension during the 60-day period preceding the laboratory claim date was present for 36.5% of claims. Diabetes (20.5%), coronary heart disease (14.4%), and chronic obstructive pulmonary disease (10.5%) were also observed more frequently than other conditions. Outpatient pharmacy claims for medications known to cause hyponatremia were observed within the -60/+15-day period before or after the laboratory date in 1 of every 4 claims (Table 1).

Compared to claims without a corresponding diagnosis code, mean age was greater among outpatient laboratory claims indicating hyponatremia with an outpatient professional diagnosis code for hyponatremia observed within 15 days (positive test, 67 versus 59 years; *p *< .001), and claims were observed significantly more often for women (61% versus 66%; *p *= < .001). Evidence of hypertension was observed nearly twice as often during the period preceding a laboratory claim with a positive test than for claims with a negative test or with no temporally adjacent outpatient professional claims (59% versus 36%; *p *< .001). Compared to claims that did not have a diagnosis code for hyponatremia, claims with a positive test were also significantly more likely to be observed for members diagnosed with kidney disease, cardiovascular conditions, and/or chronic obstructive pulmonary disease; however, claims with a positive test were significantly less likely to be observed among members with diabetes or dementia (Table 1).

Sensitivity for hyponatremia defined as serum sodium < 136 mmol/L was 3.5%; specificity was greater than 99%. Positive predictive value was 63%, and negative predictive value was 98% (Table 2). Sensitivity values for the ≤ 133 mmol/L, ≤ 130 mmol/L, and ≤ 125 mmol/L strata were 7.5%, 13.9%, and 29.6%, respectively. Specificity was greater than 99% for all of the alternative cutoff points (Table 3).

**Table 2:** Relationship between ICD-9-CM code documentation and laboratory serum sodium < 136 mmol/L

		Hyponatremia	
		+	-
ICD-9 = 276.1	+	1407	839
	-	39,261	1,859,747

Sensitivity = 3.46%

Specificity = 99.95%

Positive predictive value = 62.64%

Negative predictive value = 97.93%

**Table 3:** Validity measures by laboratory serum sodium values

	Serum Sodium (mmol/L)			
	
	< 136	≤ 133	≤ 130	≤ 125
Sensitivity	3.46	7.50	13.85	29.57
False-negative rate	96.54	92.50	86.15	70.43
Specificity	99.95	99.94	99.92	99.90
False-positive rate	0.05	0.06	0.08	0.10
Positive predictive value	62.64	48.74	30.00	10.42
Negative predictive value	97.93	99.28	99.78	99.97

In the multivariable analysis exploring the relationships between undocumented hyponatremia and member demographic characteristics and comorbidities, laboratory results indicating hyponatremia among older members were significantly more likely to have a corresponding outpatient professional claim than those observed for younger members. Controlling for other comorbid diagnoses, medications known to cause hyponatremia, and sex, an increase of 10 years in age was associated with an almost 30% drop in the likelihood of a negative claim or no claim at all (odds ratio: 0.74; 95% confidence interval: 0.66, 0.74; data not shown). Laboratory claims preceded by a comorbid diagnosis of cerebrovascular disease, chronic obstructive pulmonary disease, hypertension, dementia, nephritis/nephrosis, or syndrome of inappropriate antidiuretic hormone were also less likely to be undocumented by a diagnosis code on an outpatient professional claim. Laboratory claims preceded by a comorbid diagnosis of diabetes or peripheral vascular disease were significantly more likely to be undocumented by a diagnosis code. Claims for medications known to cause hyponatremia did not have a significant impact on ICD-9-CM documentation of laboratory-identified hyponatremia (Table 4).

**Table 4:** Likelihood of a negative test for claims indicating hyponatremia*

	OR	95% CI
Age in years	0.97	0.96, 0.97
Female	0.75	0.67, 0.85
Underlying conditions and comorbidities		
Cerebrovascular disease	0.61	0.50, 0.73
Chronic obstructive pulmonary disease	0.62	0.54, 0.72
Congestive heart failure	1.09	0.89, 1.34
Coronary heart disease	0.98	0.84, 1.14
Dementia	0.44	0.27, 0.70
Diabetes mellitus	1.47	1.27, 1.70
Hypertension	0.53	0.47, 0.59
Kidney disease	1.24	0.84, 1.83
Liver cirrhosis†	0.31	0.07, 1.38
Metastatic carcinoma	1.11	0.87, 1.41
Nephritis, nephrotic syndrome, and nephrosis	0.49	0.35, 0.69
Peripheral vascular disease	1.40	1.08, 1.82
Rheumatic disease	1.27	0.91, 1.77
Syndrome of inappropriate antidiuretic hormone	0.06	0.04, 0.08
Medications known to cause hyponatremia	1.08	0.95, 1.22

* Hyponatremia was defined as serum sodium < 136 mmol/L. A negative test was defined as the presence of an outpatient professional claim with no ICD-9-CM code for hyponatremia or the absence of any outpatient professional claims within 15 days before or after the laboratory claim indicating hyponatremia.

† Indicates that the condition was present in < 1% of the population.

Abbreviations: OR indicates odds ratio; and CI, confidence interval.

## Discussion

We examined the validity of ICD-9-CM diagnosis codes for the identification of hyponatremia in an outpatient managed care population using data from the IHCIS National Managed Care Benchmark Database. Our results show that while the ICD-9-CM code for hyponatremia is highly specific in outpatient claims, its sensitivity is extremely low. Even for the most severe cases (serum sodium ≤ 125 mmol/L), we found sensitivity to be less than 30%. Similarly, the positive predictive value of an outpatient professional claim for hyponatremia was only 63% using the least strict serum sodium measurement (< 136 mmol/L). These findings are consistent with an earlier study by Movig et al [29], which showed low sensitivity for the coding of hyponatremia in inpatient settings, although the positive predictive value of ICD-9-CM codes in outpatient claims in our study was significantly less than the corresponding inpatient rates found in the previous study.

These low rates of coding for hyponatremia may be largely due to the ICD-9-CM diagnostic coding and reporting guidelines for outpatient services. According to these guidelines, only conditions that "require or affect patient care treatment or management" should be documented. Moreover, "related signs and symptoms" should not be coded when a more definitive diagnosis is known [36]. Thus, at mildly decreased levels requiring no medical intervention and/or in the presence of causal underlying disease such as syndrome of inappropriate antidiuretic hormone or liver cirrhosis, it may be inappropriate to code for hyponatremia on an outpatient claim. Similarly, there may be limited space available for diagnoses on the outpatient claim form. The IHCIS database, for example, allows for a maximum of 3 diagnosis codes on each professional claim record. As shown in Table 1, hyponatremia is often seen in the presence of other significant comorbidities. Given that ICD-9-CM coding guidelines also specify that one should "list first the ICD-9-CM code for the diagnosis, condition, problem, or other reason for encounter/visit shown in the medical record to be chiefly responsible for the services provided" [36], other conditions may have taken precedence on the claim form.

The clinical consequences of even mild hyponatremia are well-documented [3,4,6,34,35,37-39]. In a cohort of elderly patients, hyponatremia at hospital admission was a significant independent predictor of mortality after adjustment for age, sex, length of stay, and several clinical factors [7]. Hyponatremia (serum sodium ≤ 135 mmol/L) is independently associated with major complications, greater length of stay, higher hospital costs, and greater inpatient mortality in patients with suspected congestive heart failure [1] and with greater in-hospital and 60-day mortality in patients with heart failure [2]. It is an independent predictor of 30-day mortality in patients with acute ST-segment elevation myocardial infarction [3]. Given the preponderance of evidence suggesting that hyponatremia is an independent predictor of poorer outcomes, our findings suggest that the prognostic value of even severe hyponatremia may be underappreciated.

Another potential explanation for the low observed rates of coding is that hyponatremia, as identified by a single laboratory value, may have been a transient condition for some members. We analyzed single laboratory values and did not consider the results of prior or subsequent testing. Thus, it is possible that some members received follow-up testing that showed the condition to be resolved, thereby eliminating the need for documentation on the outpatient professional claim. Also, because the sample included commercially insured adults with relatively low amounts of comorbid illness, mildly decreased levels of serum sodium may not be clinically significant or require medical intervention. Nevertheless, we found sensitivity to be less than 30%, even in the case of serum sodium ≤ 125 mmol/L. Finally, because documentation of hyponatremia is unlikely to generate additional reimbursement for outpatient services, there may be little incentive for physicians to include it on the claim.

In contrast, the positive predictive value of outpatient claims for hyponatremia was also low (63% for serum sodium < 136 mmol/L). This finding may reflect the fact that hyponatremia is a chronic condition for some patients and is therefore likely to be noted on outpatient professional claims despite the fact that there are no observable laboratory claims within +/-15 days to support the diagnosis. The data used here, however, do not include sufficient detail to validate this hypothesis.

Although the impact of medications known to cause hyponatremia was not significant in the multivariable model, there was a trend toward less ICD-9-CM documentation of laboratory-identified hyponatremia in the presence of this factor. Outpatient claims for medications known to cause hyponatremia were observed within 60 days before through 15 days after 25% of all laboratory claims indicating hyponatremia. This finding suggests that some clinicians may choose not to code hyponatremia when it may be the result of drug therapy. Other results of the multivariable model also suggest that ICD-9-CM coding of laboratory-identified hyponatremia may be especially poor for patients with peripheral vascular disease or diabetes. Claims for members with these conditions were more than 40% less likely to have an ICD-9-CM code for hyponatremia.

This study has some limitations. First, outpatient laboratory data are only available for approximately 10% of members in the IHCIS database. Second, the IHCIS database consists of managed care claims only and is therefore representative of an employer-based, commercially insured population. We expect that the elderly are significantly underrepresented in the database. Because the risk of hyponatremia is highest among the elderly and our results show that the frequency of coding for hyponatremia increases with age, our findings may not be generalizable to older populations. Inconsistent and inaccurate coding and the absence of clinical data regarding disease severity may have also affected our estimates. We required just 60 days of continuous coverage before and 15 days of coverage after the reference serum sodium date and searched for evidence of comorbidity claims within that time period only. Although this approach maximized our sample size, it also limited the available time frame in which we could observe comorbid illnesses, which may have led to an underestimation of these conditions in our analyses. We also chose to search for outpatient professional claims that were temporally adjacent to serum sodium laboratory tests (+/-15 days). As a result, the analysis is unable to capture instances where abnormal test results were addressed by providers without generation of an outpatient claim (eg, by phone or e-mail) or when a follow-up visit was performed outside of this time window. Finally, the study used outpatient clinical and laboratory information only. The results do not account for the possibility that a laboratory-identified diagnosis of hyponatremia, for example, may have been documented by an ICD-9-CM code on an inpatient claim just before or after the outpatient laboratory date.

## Conclusion

Our results suggest that the use of ICD-9-CM codes in administrative data alone is insufficient to identify hyponatremia in outpatient populations. Whenever possible, supplementary laboratory information should be used to help overcome this limitation of administrative claims.

## Abbreviations

CI: confidence interval; ICD-9-CM: *International Classification of Diseases, Ninth Revision, Clinical Modification*; IHCIS: Integrated Healthcare Information Services; OR: odds ratio.

## Competing interests

Ms Shea declares that she has no competing interests.

Dr Curtis reports receiving research and salary support from Allergan Pharmaceuticals, GlaxoSmithKline, Lilly, Medtronic, Novartis, Ortho Biotech, OSI Eyetech, Pfizer, and Sanofi-Aventis.

Dr Szczech reports receiving personal income for consulting from Sanofi-Aventis.

Dr Schulman reports receiving research and/or salary support from Actelion, Allergan, Amgen, Arthritis Foundation, Astellas Pharma, Bristol-Myers Squibb, The Duke Endowment, Genentech, Inspire Pharmaceuticals, Johnson & Johnson, Kureha Corporation, LifeMasters Supported SelfCare, Medtronic, Merck, Nabi Biopharmaceuticals, National Patient Advocate Foundation, North Carolina Biotechnology Center, Novartis, OSI Eyetech, Pfizer, Roche, Sanofi-Aventis, Schering-Plough, Scios, Tengion, Theravance, Thomson Healthcare, Vertex Pharmaceuticals, Wyeth, and Yamanouchi USA Foundation; receiving personal income for consulting from Avalere Health, LifeMasters Supported SelfCare, McKinsey & Company, and the National Pharmaceutical Council; having equity in and serving on the board of directors of Cancer Consultants; having equity in and serving on the executive board of Faculty Connection LLC; and having equity in Alnylam Pharmaceuticals.

## Authors' contributions

AMS obtained the data, performed the statistical analysis, analyzed and interpreted the data, and drafted the manuscript. LHC conceived and designed the study, obtained the data, analyzed and interpreted the data, obtained funding, supervised the study, and revised the manuscript for important intellectual content. LAS provided expertise in nephrology, analyzed and interpreted the data, and revised the manuscript for important intellectual content. KAS conceived and designed the study, analyzed and interpreted the data, obtained funding, and revised the manuscript for important intellectual content. All authors read and approved the final manuscript.

## Pre-publication history

The pre-publication history for this paper can be accessed here:


